# Single-Use vs. Reusable Digital Flexible Ureteroscope to Treat Upper Urinary Calculi: A Propensity-Score Matching Analysis

**DOI:** 10.3389/fsurg.2021.778157

**Published:** 2022-01-10

**Authors:** Fang Huang, Xiaoqiong Zhang, Yu Cui, Zewu Zhu, Yongchao Li, Jinbo Chen, Feng Zeng, Yang Li, Zhiyong Chen, Hequn Chen

**Affiliations:** ^1^Department of Urology, Xiangya Hospital, Central South University, Changsha, China; ^2^National Clinical Research Center for Geriatric Disorders, Xiangya Hospital, Central South University, Changsha, China; ^3^Department of Transplantation, Xiangya Hospital, Central South University, Changsha, China

**Keywords:** upper urinary calculi, single-use, flexible ureteroscope, treatment outcomes, cost analysis

## Abstract

**Objective:** The purpose of this research was to compare the treatment outcomes and costs of a single-use and reusable digital flexible ureteroscope for upper urinary calculi.

**Methods:** Four hundred forty patients with reusable digital flexible ureteroscope and 151 patients with single-use flexible digital ureteroscope were included in this study. Through exclusion and inclusion criteria and 1:1 propensity-score matching analysis based on baseline characteristics, ultimately, 238 patients (119:119) were compared in terms of treatment outcomes. The cost analysis was based on the costs of purchase, repair, and reprocessing divided by the number of all procedures in each group (450 procedures with reusable digital flexible ureteroscope and 160 procedures with single-use digital flexible ureteroscope).

**Results:** There was no statistical significance in mean operation time (*P* = 0.666). The single-use digital flexible ureteroscope group has a shorter mean length of hospital stay than the reusable digital flexible ureteroscope group (*P* = 0.026). And the two groups have a similar incidence of postoperative complications (*P* = 0.678). No significant difference was observed in the final stone-free rate (*P* = 0.599) and the probability of secondary lithotripsy (*P* = 0.811) between the two groups. After 275 procedures, the total costs of a single-use flexible ureteroscope would exceed the reusable flexible ureteroscope.

**Conclusion:** Our data demonstrated that the single-use digital flexible ureteroscope is an alternative to reusable digital flexible ureteroscopy in terms of surgical efficacy and safety for upper urinary calculi. In terms of the economics of the two types of equipment, institutions should consider their financial situation, the number of FURS procedures, the volume of the patient's calculus, surgeon experience, and local dealerships' annual maintenance contract when making the choice.

## Introduction

Urolithiasis is a common urological disease and its incidence has been increasing globally in recent years (1). With the progress of modern medicine, flexible ureteroscopic (FURS) lithotripsy has become the main surgical management to treat upper urinary calculi smaller than 2 cm (2), as it can pass through the natural lumen to the renal cavities and stone-free rates (SFR) ranged between 80 and 90% (3). However, there are intractable deficiencies that limit the widespread use of reusable FURS in countries with restricted healthcare expenditures, including high purchase and maintenance costs (4). In addition, reusable FURS disinfection requires specialized equipment and personnel, which increases costs and risks of cross-infection due to disinfection failure (5). Given these deficiencies, single-use FURS have been developed in recent years, which are exempt from disinfection and maintenance. Currently, several single-use devices such as LithoVue^TM^ (Boston Scientific, Natick, MA), Uscope^TM^ (Zhuhai Pusen Medical Technology Co. Ltd., Zhuhai, China), NeoFlex^TM^ (Neoscope; Inc, San Jose, CA), and ZebraScope^TM^ (Happiness Works Medical Technology Co, LTD, Beijing, China) are available. Preliminary studies indicated that single-use FURS can be as effective and safe as reusable FURS (6, 7) and may be cost beneficial by eliminating the expensive reprocessing and repair costs in certain circumstances (8, 9). But we still lack official recommendations and reliable evidence (10).

Therefore, the objective of this study mainly concerns the clinical performance and costs of a single-use digital FURS (ZebraScope^TM^) compared with a reusable digital FURS (URF-V; Olympus, Tokyo, Japan).

## Methods

### Patients and Data Collection

**Figure 1:**
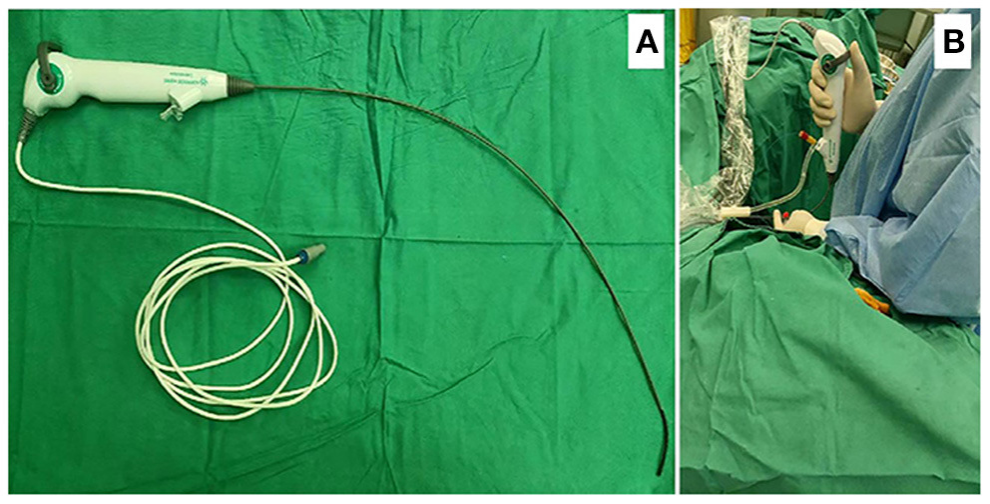
**(A)** Chinese single-use FURS ZebraScopeTM (Happiness Workshop): The outer diameter of the front end of the lens is F7.4, and the maximum outer diameter of the lens is F8.6. The operating channel is a single channel with an inner diameter of F3.6. The steering angle of the mirror head end is 1:1, and the minimum bending radius is about 8 mm. The head end can bend more than 270 in both no-load. **(B)** Application of the single-use digital FURS ZebraScopeTM during operation.

This study protocol was approved by the Ethics Committee of the Xiangya Hospital, Central South University. Four hundred forty patients (10 patients underwent two lithotripsy procedures) were treated with reusable digital FURS (between January 2018 and February 2020) and 151 patients (nine patients underwent two lithotripsy procedures) were treated with single-use digital FURS (between March 2020 and September 2020) for upper urinary calculi and their charts were retrospectively reviewed. All procedures were performed by experienced surgeons at our medical center and the course of the surgery is described in the Surgical Technique (the single-use digital FURS as shown in Figure 1). The inclusion criteria were as follows: (1) patients age ≥18 years old; and (2) patients were treated with reusable digital FURS and single-use digital FURS for upper urinary calculi. According to the following exclusion criteria: (1) patients age <18 years old; (2) patients undergoing bilateral procedures or simultaneously combined with other surgery; (3) patients with special situations such as pregnancy, duplicate ureteral deformity and horseshoe kidney, 408 patients and 142 patients were enrolled in single-use FURS and reusable FURS group respectively for treatment outcomes analysis. Subsequently, through 1:1 propensity-score matching analysis based on age, sex, body mass index (BMI), American Society of Anesthesiologists classification (ASA), stone hardness, stone burden, stone location, ureteric stent implanted preoperatively, positive preoperative urine culture, solitary kidney stone, procedural laterality, history of ipsilateral urolithiasis surgery, and degree of hydronephrosis, ultimately, 238 patients (119:119) in the two groups were compared in terms of treatment outcomes. All procedures (450 procedures with reusable FURS and 160 procedures with single-use FURS) were reviewed for costs analysis.

All patients underwent an abdominal non-contrast computed tomography (CT) scan preoperatively to evaluate the stone size, position, and hardness (measured in Hounsfield units, HU). Plain film of kidney-ureter-bladder (KUB) was performed to evaluate stone-free status at 1 day and 1 month postoperatively, and CT will be performed again only when patients need a secondary lithotrity. The demographic variables, operation time, length of hospital stay, postoperative complications, and other clinical data were collected through our electronic medical record system. The urinary microbial culture was performed in all patients 1 week before surgery. Any patient with a positive culture was given sensitive antimicrobial therapy preoperatively based on antibiotic sensitivity tests and well-controlled urinary tract infections were confirmed by urinary cultures before surgical intervention. Patients with negative urine culture received intravenous antimicrobial (Cefuroxime) prophylaxis 30 min before the anesthetic.

### Surgical Technique

Patients were placed in the lithotomy position after general anesthesia. Under the guidance of 4F ureteral catheters, a 9.8-F semirigid ureteroscope (URS) (Karl Storz, Germany) was placed into the ureter to detect whether there was stenosis or abnormality and to dilate the ureter to facilitate the placement of a ureteral access sheath (UAS). Subsequently, a Zebra guidewire was inserted into the ureter through the URS. Then, the URS was removed and a 12/14-Fr Flexor UAS (Cook Urology, 45 cm for male, 35 cm for female) was advanced into renal pelvis directed by the guidewire (If UAS implantation failed, double J tubes were implanted in the first stage, and the second procedure was performed 2 weeks later). Subsequently, the 8.6-F single-use digital FURS ZebraScope^TM^ (Figure 1) or 9.9-F reusable digital FURS (URF-V; Olympus, Tokyo, Japan) was placed into the pelvis through the UAS. Lithotripsy was performed using holmium:yttrium-aluminum-garnet laser (Ho: YAG) with a 200-μm fiber at an output power of 50–60 W and a frequency level of 15–24 Hz. The rubble fragments were recovered using a 2.4F zero-tip Nitinol stone basket (Cook Medical, Bloomington, IN, USA). After repeated examination of the collection system, it was confirmed that the stones were completely broken and removed. The operation ended with the placement of a double J stent in the ureter for drainage for 1 month.

### Clinical Outcomes Analysis

The extent of hydronephrosis was assessed according to the Society of Fetal Urology grading system (11, 12). Postoperative complications were evaluated according to the Clavien-Dindo classification system (13). Septic shock was defined according to the third international consensus (14). Stone volume was calculated using the following formula (0.785 × length_max_ × width_max_) according to CROES (15), and the burden of multiple stones was calculated as the sum of the volume of all stones. Postoperative stone-free status was defined as the absence of stone fragment > 3 mm on KUB. According to the size and location of residual stones, two experienced professors comprehensively evaluated whether retreatment was needed (CT will be performed only when patients need secondary lithotripsy). In addition, medical images of all patients were independently read by a radiologist and a urologist to measure the calculi burden as determined by CT and to evaluate calculi-free status as determined by KUB after surgery. The clinical outcomes of patients who received their first treatment with FURS lithotripsy during this treatment period were evaluated.

### Crude Cost Analysis

As this was a retrospective study, we were unable to balance the preoperative characteristics in the cost analysis. We performed a crude cost analysis for all procedures undergoing FURS lithotripsy during this study period. All costs were presented in dollars ($) (One dollar is ~6.541yuan). Two reusable FURS were available in our institution which were purchased at market price in 2016 and 2017 respectively. Due to those devices were not new at the time of the study, and we could not count the number of procedures performed before the study. The original purchase costs of the two sets of reusable equipment were modeled as residual value by annual depreciation rate (Approximately $275220; 1800000yuan). Between January 2018 and February 2020, the reusable FURS conducted six repairs at a cost of ~$183480 (1200000yuan). Extrapolating from the data provided by the Disinfection supply center in our hospital, reprocessing costs were ~$80 (523yuan) per procedure, which included the costs of inspection, pre-cleaning, decontamination, assembly, and sterilization. Purchasing prices of disinfection equipment have been left out in our study. The personnel cost was about $40 (262yuan) per procedure based on the hourly wage of the central disinfection technician combined with the average approximate time to reprocess FURS. According to the present local market price, the cost of single-use FURS was about $1529 (10000yuan) per procedure. The total costs were estimated based on the following equations which are similar to the provided by Martin et al. (9).


$$\begin{array}{r} Total\;costs\;of\;single\;-\;use\;FURS\;(costs\;of\;single\\ -\;use\;FURS\;per\;procedure)\;\times X,\\ where\;X=\;number\;of\;procedures\\ Total\;costs\;of\;reusable\;FURS=(Original\;purchasing\\ cost\;of\;reusable\;FURS)+\\ {}[(repair\;cost\;per\;procedure)+\\ \left(Reprocessing\;cost\;per\;procedure\right)\\ +(labor\;cost\;per\;procedure)]\times \\ where\;X=number\;of\;procedures \end{array}$$

The cost per procedure was the total costs divided by the number of procedures. Assuming that the maintenance cost per procedure is roughly constant over a long period (Excluding the possible increase in the number of repairs due to aging of FURS), from the above two equations, we can also get a formula that can help the institution to calculate the number of operations performed when the total costs of the two devices reach the equilibrium point.


$$\begin{array}{r} Y=Original\;purchasing\;costs\;of\;reusable\;FURS÷\\ (cost\;of\;single-use\;FURS\;per\;procedure-\;the\\ average\;maintenace\\ costs\;of\;reusable\;FURS\;per\;procedure)\\ where=Y\;the\;equilibrim\;point\;of\;procedure\;volumes \end{array}$$

### Statistical Methods

Chi-square test or Fisher's exact test was used to analyze the proportion of categorical variables; Student's *t*-test was used to analyze numerical variables with normal distribution. A two-sided *P*-value less than or equal to 0.05 was considered statistically significant. The logistic regression model was used to calculate the propensity score of each research object for 1:1 propensity-score matching analysis. Statistical analysis and 1:1 propensity-score matching analysis were performed using the Statistical Package for the Social Sciences 22.0 (SPSS for Windows, Chicago, IL, USA).

## Results

### Clinical Outcomes Analysis

Preoperative clinical data of the two groups for treatment outcomes analysis (408 vs. 142) are shown in Table 1. After 1:1 propensity-score matching analysis, baseline characteristics of those patients were evenly distributed in two groups (Table 1).

**Table 1:** Baseline characteristics of the included patients for clinical outcomes analysis.

**Parameters**	**Before propensity-score matching**			**After propensity-score matching**		
	**Reusable** **N (408)**	**Single-use** **N (142)**	***P*** **-value**	**Reusable** **N (119)**	**Single use** **N (119)**	***P*-value**
Age (years), mean ± SD	52.0 ± 12.3	49.4 ± 12.9	0.030^a^	49.0 ± 12.0	49.4 ± 12.7	0.821^a^
Gender, n (%)						
Male	258 (63.2%)	93 (65.5%)	0.630^b^	77 (64.7%)	79 (66.4%)	0.785^b^
Female	150 (36.8%)	49 (34.5%)		42 (35.3%)	40 (33.6%)	
BMI (kg/m^2^), mean ± SD	23.8 ± 3.1	24.1 ± 3.7	0.255^a^	24.2 ± 3.1	24.0 ± 3.4	0.620^a^
Pre-stented, n (%)	60 (14.7%)	19 (13.4%)	0.698^b^	16 (13.4%)	16 (13.4%)	1.000^b^
Positive preoperative urine culture, n (%)	38 (9.3%)	18 (12.7%)	0.254^b^	15 (12.6%)	18 (15.1%)	0.574^b^
Solitary kidney stone, n (%)	50 (12.3%)	14 (9.9%)	0.443^b^	12 (10.1%)	12 (10.1)	1.000^b^
Procedural laterality, n (%)						
Left	215 (52.7%)	71 (50.0%)	0.580^b^	59 (49.6%)	58 (48.7%)	0.897^b^
Right	193 (47.3%)	71 (50.0%)		60 (50.4%)	61 (51.3%)	
History of Ipsilateral urolithiasis surgery, n (%)			0.961^c^			0.973^c^
None	292 (71.6%)	100 (70.4%)		86 (72.3%)	86 (72.3%)	
PCNL	37 (9.1%)	15 (10.6%)		11 (9.2%)	11 (9.2%)	
RIRS or URL	54 (13.2%)	20 (14.1%)		15 (12.6%)	17 (14.3%)	
EWSL	19 (4.7%)	5 (3.5%)		5 (4.2%)	3 (2.5%)	
Open operation	6 (1.5%)	2 (1.4%)		2 (1.7%)	2 (1.7%)	
ASA, n (%)						
Class 1 and 2	304 (74.5%)	111 (78.2%)	0.383^b^	94 (79.0%)	94 (79.0%)	1.000^b^
Class 3 and 4	104 (25.5%)	31 (21.8%)		25 (21.0%)	25 (21.0%)	
Degree of hydronephrosis, n (%)						
None or mild	380 (93.1%)	128 (90.1%)	0.247^b^	107 (89.9%)	110 (92.4)	0.493^b^
Moderate or severe	28 (6.9%)	14 (9.9%)		12 (10.1%)	9 (7.6%)	
Stone characteristics						
Stone hardness (HU), mean ± SD	1000 ± 261	976 ± 260	0.340^a^	964 ± 240	972 ± 257	0.787^a^
Stone burden (cm^2^), mean ± SD	59.9 ± 39.5	71.4 ± 38.9	0.003^b^	69.3 ± 37.1	69.5 ± 34.5	0.970^b^
Stone localization, n (%)			0.560^c^			0.958^c^
Upper segment of ureter	158 (38.7%)	57 (40.1%)		46 (38.7%)	49 (41.2%)	
Upper calix	12 (2.9%)	4 (2.8%)		4 (3.4%)	4 (3.4%)	
Middle calix	30 (7.4%)	6 (4.2%)		6 (5.0%)	6 (5.0%)	
Lower calix	37 (9.1%)	17 (12.0%)		13 (10.9%)	16 (13.4%)	
Pelvis	55 (13.5%)	12 (8.5%)		16 (13.4%)	11 (9.2%)	
Upper ureteral segment with pelvis or calices	67 (16.4%)	28 (19.7%)		16 (13.4%)	18 (15.1)	
Pelvis with calices	23 (5.6%)	7 (4.9%)		9 (7.6%)	6 (5.0%)	
Multiple calices	26 (6.4%)	11 (7.7%)		9 (7.6%)	9 (7.6%)	
Comorbidities, n (%)			0.212^b^			0.225^b^
None	264 (64.7%)	97 (68.3%)		72 (60.5%)	83 (69.7%)	
Diabetes mellitus	75 (18.4%)	16 (11.3%)		9 (7.6%)	9 (7.6%)	
Hypertension	16 (3.9%)	9 (6.3%)		24 (20.2%)	12 (10.1%)	
Renal insufficiency	15 (3.7%)	8 (5.6%)		8 (6.7%)	6 (5.0%)	
Multi-comorbidities^d^	38 (9.3%)	12 (8.5%)		6 (5.0%)	9 (7.6%)	

*^a^Continuous variable were assessed by t-test.*

*^b^Chi-square test.*

*^c^Fisher's exact test (two-tailed).*

*^d^Patients with two or more comorbidities*.

The treatment outcomes with two surgical devices are shown in Table 2. There was no significant difference in the mean operative time between the two groups (60.43 ± 22.76 vs. 61.61 ± 19.36 min, *P* = 0.666). The mean length of hospital stay in the single-use FURS group was significantly shorter than that in the reusable FURS group (6.86 ± 1.82 days vs. 7.42 ± 2.06 days, *P* = 0.026), but there was no significant difference in postoperative length of hospital stay between the two groups (2.64 ± 1.32 vs. 2.81 ± 1.55 days, *P* = 0.368). The average decrease of hemoglobin (Hb) (*P* = 0.224) and hematocrit (Hct) (*P* = 0.345) was also no significant difference between the two groups.

**Table 2:** Treatment outcomes of the reusable FURS group and the single-use FURS group.

**Surgical outcomes**	**Reusable**	**Single-use**	***P*-value**
Decline in Hb level (g/L)	3.74 ± 7.42	2.39 ± 9.46	0.224^b^
Decline in Hct level (%)	1.27 ± 2.48	0.91 ± 3.27	0.345^b^
Operative time (min), mean ± SD	60.43 ± 22.76	61.61 ± 19.36	0.666^b^
Hospital stays (days), mean ± SD	7.42 ± 2.06	6.86 ± 1.82	0.026^b^
Postoperative hospital stays (days), mean ± SD	2.81 ± 1.55	2.64 ± 1.32	0.368^b^
Initial SFR (1 day after surgery), n (%)	90 (75.6%)	93 (78.2%)	0.645^c^
Final SFR (1 month after surgery), n (%)	98 (82.4%)	101 (84.9%)	0.599^c^
Re-operation of the stone, n (%)	10 (8.4%)	9 (7.6%)	0.811^c^
Total complications^a^ [Clavien grade classification, n (%)]	12 (10.1%)	14 (11.8%)	0.678^c^
Grade I	6 (5.0%)	9 (7.5%)	0.424^c^
Simple fever^e^	2 (1.7%)	3 (2.5%)	
Flank pain	2 (1.7%)	2 (1.7%)	
Nausea	1 (0.8%)	3 (2.5%)	
Fever and flank pain	1 (0.8%)	1 (0.8%)	
Grade II	3 (2.5%)	4 (3.4%)	1.00^d^
Urosepsis requiring only additional antibiotics	3 (2.5%)	4 (3.4%)	
Grade III	1 (0.8%)	0 (0%)	1.00^d^
Steinstrasse requiring surgical treatment	1 (0.8%)	0 (0%)	
Grade IV	2 (1.7%)	1 (0.8%)	1.00^d^
Septic shock	2 (1.7%)	1 (0.8%)	
Infection-related complications (moderate to severe) ^f^	5 (4.2%)	5 (4.2%)	1.00^d^

*^a^Patients with multiple complications are finally classified according to the most severe one.*

*^b^Continuous variables were assessed by t-test.*

*^c^Chi-square test.*

*^d^Fisher's exact test (two-tailed).*

*^e^Fever patients only need antipyretic drugs or physical hypothermia therapy.*

*^f^Patients with Urosepsis or Septic shock*.

The two groups experienced similar rates of overall postoperative complications (10.1% vs. 11.8 %, *P* = 0.678). The single-use group was associated with a higher incidence of grade I complication (7.5% vs. 5.0%, *P* = 0.424) than the reusable group, but it had no statistical difference. Urosepsis requiring only additional antibiotics was the main grade II complication and occurred no statistically different incidence rates in the two groups (2.5% vs. 3.4%, *P* = 1.0). Only one patient in the reusable FURS group developed steinstrasse after discharge and underwent surgery (Grade III). Septic shock (Grade IV) was observed in 2 (1.7%) and 1 (0.8%) patients in the reusable and single-use groups, respectively (*P* = 1.00). There was also no significant difference in moderate to severe infection-related complications (4.2% vs. 4.2%, *P* = 1.00).

Initial SFR of the reusable FURS group and single-use FURS groups were 75.6% and 78.2% (*P* = 0.645). There was also no significant difference in final SFR between the two groups (82.4% vs. 84.9%, *P* = 0.599). And there were 10 patients (8.4%) in the reusable FURS group and 9 patients (7.6%) in the single-use FURS group who required repeated surgery to remove residual stones (*P* = 0.811).

### Crude Cost Analysis

**Figure 2:**
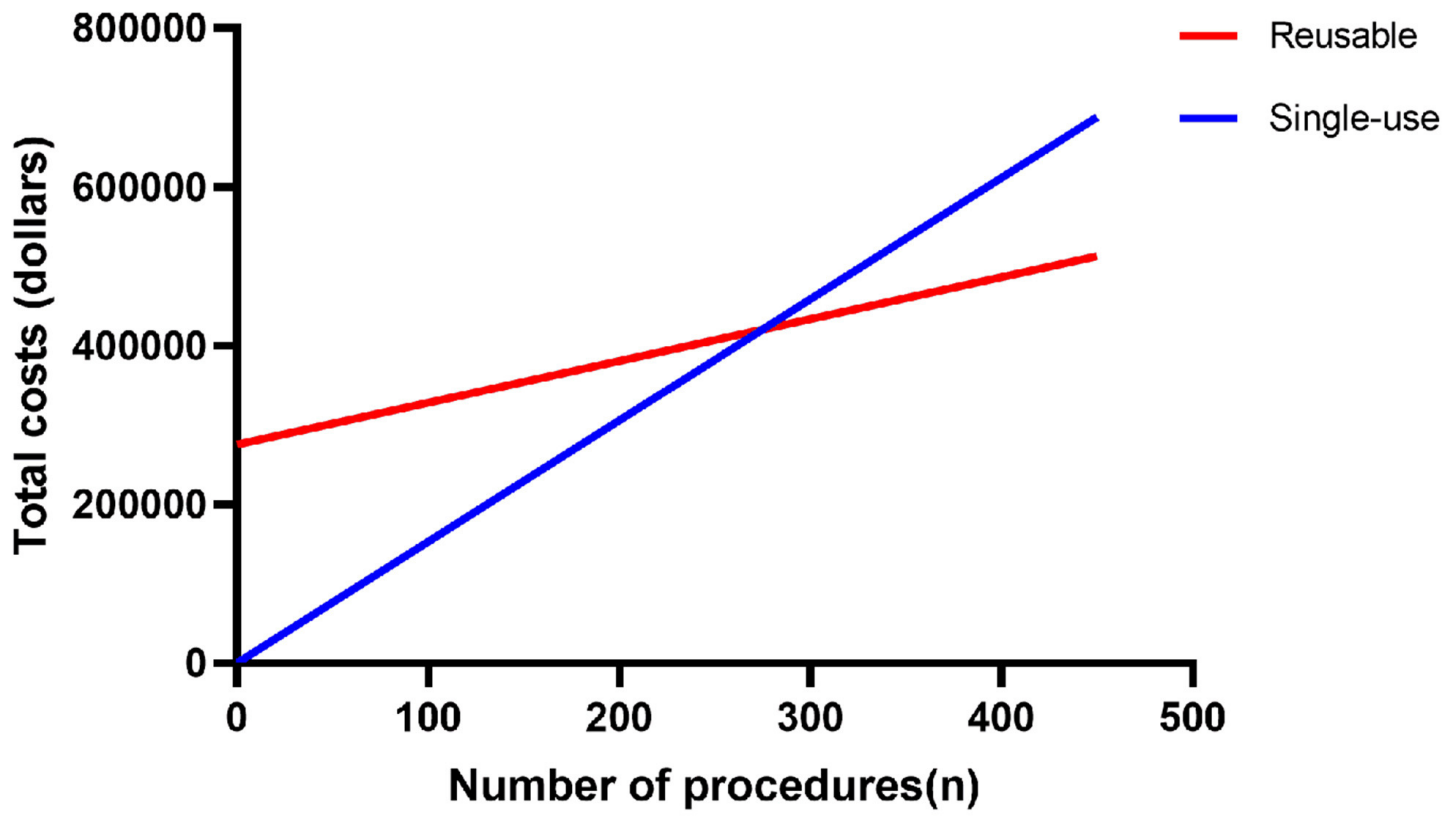
The linear graphs demonstrate the change in total costs of reusable FURS and single-use FURS as the number of procedures increases.

The costs of reusable FURS or single-use FURS per procedure are shown in Table 3. Between January 2018 and February 2020, the repair cost per procedure is about $408 (2668yuan) for reusable FURS. After the original purchasing costs, the average cost per reusable FURS was ~$528 (2453yuan). When taking into account original purchasing costs, we should consider the impact of procedure volume on the final cost per procedure, which will decrease with the increase of procedure volume. The cost per single-use FURS was ~$1529 (10000yuan). According to our formula, the break-even point between the two alternatives appears to be 275 procedures in our institution. Total costs or cost per procedure of single-use FURS would exceed the reusable FURS after 275 procedures as shown in Figure 2.

**Table 3:** The costs of reusable FURS or single-use FURS per procedure.

**Cost items**	**Reusable FURS per case; dollars (Renminbi)**	**Single-use FURS per case; dollars (Renminbi)**
Original purchasing cost	275220/X	1529 (10000yuan)
Repair cost	408 (2668 yuan)	0
Reprocessing cost	80 (523yuan)	0
Personnel cost	40 (262yuan)	0
Total cost	275220/X +528	1529 (10000yuan)

*X, number of procedures*.

## Discussion

With the rapid development of endoscopic surgical equipment, the single-use FURS, which are designed to alleviate the deficiencies of high cost and recurrent damage associated with the use of reusable FURS, gradually come to the attention of our urologists. Some prospective clinical studies have shown that some kind of single-use FURS has comparable performance to reusable FURS (7, 16–19). However, there are many types of single-use FURS on the market at present, and more studies are needed to further confirm their value in clinical application. Additionally, there is a scarcity of retrospective clinical data about the comparison between single-use FURS and reusable FURS.

In this study, through the propensity-score matching analysis, we retrospectively compared the clinical outcomes of 238 patients who experienced single-use FURS or reusable FURS lithotripsy. The results showed that the two devices performed similarly in terms of surgical efficacy and safety, similar to a prospective multicenter randomized controlled trial that compared the clinical outcomes of single-use digital FURS (ZebraScope^TM^) and reusable digital FURS (URF-V) (17). But a study about single-use digital FURS (LithoVue^TM^) vs. reusable fiberoptic FURS (URF-P6) showed that the performance of single-use FURS was better than reusable FURS in terms of mean operative time and surgical complications (19). The reason for the different results may be that digital FURS, compared with the fiberoptic FURS, has clearer images and a wider viewing angle (20, 21). There is no consensus in the operative time between single-use FURS and reusable FURS. Although several studies have found that the single-use FURS have the advantage of shorter surgical time (22–24), a series of prospective comparative research between single-use FURS and reusable FURS have found no significant difference in mean operative time between these two surgical devices (7, 17, 18, 25). As such, a prospective study with larger sample size is needed to confirm the performance of single-use and reusable FURS in terms of operative time. In this study, overall postoperative complications of the single-use FURS and reusable FURS group were also similar (10.1% vs. 11.8 %, *P* = 0.678) and are consistent with the incidence of complications (10–15%) have been reported (7, 17, 26, 27).

It has been reported that the positive rate of pre-use ureteroscope cultures was 12.1% after sterilization (28). A single-use FURS can automatically eliminate the possibility of cross-contamination by bypassing the reprocessing and sterility steps. But ever since the revolutionary invention was used in the clinic, no postoperative cross-contamination was recorded in patients after strict compliance with disinfection protocols for ureteroscope (28). Therefore, in this study, it is reasonable to observe that there is no difference in the incidence of moderate to severe infection-related complications between the single-use FURS group and the reusable FURS group (4.2% vs. 4.2%, *P* = 1.00).

Concerning the SFR, the current study found that the performance of single-use FURS is not inferior to reusable FURS (6, 7, 17, 19). Even a pooled analysis of 772 patients who experienced single-use FURS or reusable FURS showed that single-use FURS was associated with a higher SFR (OR: 1.50; 95% CI, 1.06–2.12; *P* = 0.02) than reusable FURS (24). In the present study, to accurately evaluate the performance of the two surgical devices in SFR, we conducted a detailed classification of stone location as shown in Table 1. The result showed that the final SFR was 84.9% for the single-use FURS group and 82.4% for the reusable FURS group (*P* = 0.599). Moreover, there was no significant difference in the rate of second-stage surgical treatment of calculi. A multicenter randomized controlled trial evaluated the same single-use FURS(ZebraScope^TM^) with an SFR of 77% (17), which is lower than the present study. That may be due to the uneven skill of the surgeons involved in the multicenter study. Through the above discussion, in terms of clinical efficacy and safety, single-use digital FURS maybe be an effective and safe alternative to reusable FURS for experienced users. But given the vulnerability of reusable FURS, prioritizing the use of single-use FURS for trainees may significantly reduce the maintenance costs of reusable FURS.

It is difficult to reach a unified conclusion in cost analysis, because the total cost may vary by institution and the local price of commodities. To date, the LithoVue^TM^ is the only single-use FURS with a thorough economic analysis. A micro-costing analysis indicated that the costs per case associated with reusable and single-use ureteroscopes are comparable (29). One study showed that a single-use FURS was considerably less expensive than a reusable FURS when it is priced at 850USD (8). Some studies have shown that using single-use FURS in high-risk breakage cases (such as staghorn stones, stones located in the lower pole) is an economical choice (16, 30). In this research, After the original purchasing costs, the average cost per reusable FURS was ~$528 (2453yuan), which was lower than $799.60 per case of Martin's study (9). According to our formula, after 275 FURS procedures, the cost-benefit analysis would favor the use of reusable FURS rather than disposable ureteroscope in this hospital, but more start-up capital is needed for the reusable FURS. Thus, at current market prices for single-use FURS, institutions should choose the most suitable device for themselves based on the number of FURS procedures and their financial situation.

There are still several limitations in this study. First, this study was a retrospective single-center study. Although a 1:1 propensity-score matching was used for clinical efficacy analysis, there were still some inevitable biases that could affect the accuracy of results. Second, we have only briefly analyzed the costs of two types of equipment and were unable to balance the preoperative characteristics. Therefore, future prospective randomized studies with large case sizes are needed to confirm the current results.

## Conclusions

Our data demonstrated that the single-use FURS is an alternative to reusable FURS in terms of surgical efficacy and safety for upper urinary calculi. In terms of the economics of the two types of equipment, institutions should consider their financial situation, the number of FURS procedures, the volume of the patient's calculus, surgeon experience, and local dealerships' annual maintenance contract when making the choice.

## Data Availability Statement

The original contributions presented in the study are included in the article/supplementary material, further inquiries can be directed to the corresponding author.

## Ethics Statement

The studies involving human participants were reviewed and approved by the Ethics Committee of the Xiangya Hospital, Central South University (No: 202105087). The patients/participants provided their written informed consent to participate in this study. Written informed consent was obtained from the individual(s), and minor(s)' legal guardian/next of kin, for the publication of any potentially identifiable images or data included in this article. The authors are accountable for all aspects of the work, including ensuring that questions related to the accuracy or integrity of any part of the work have been appropriately investigated and resolved. The study was conducted following the Helsinki Declaration (as revised in 2013).

## Author Contributions

FH: conceptualization, visualization, methodology, and writing original draft. XZ and YC: validation, data curation, and investigation. ZZ, YoL, and FZ: investigation and resources. JC: writing review and editing. YaL and ZC: methodology, software, and formal analysis. HC: conceptualization, project administration, and supervision. All authors contributed to the article and approved the submitted version.

## Funding

This study was supported by the Natural Science Foundation of Hunan Province (2017JJ3482).

## Conflict of Interest

The authors declare that the research was conducted in the absence of any commercial or financial relationships that could be construed as a potential conflict of interest.

## Publisher's Note

All claims expressed in this article are solely those of the authors and do not necessarily represent those of their affiliated organizations, or those of the publisher, the editors and the reviewers. Any product that may be evaluated in this article, or claim that may be made by its manufacturer, is not guaranteed or endorsed by the publisher.

## Abbreviations

FURS: flexible ureteroscope
SFR: stone-free rates
BMI: body mass index
ASA: American Society of Anesthesiologists classification
CT: Computed tomography
HU: Hounsfield units
KUB: plain film of kidney-ureter-bladder
Hct: hematocrit
Hb: hemoglobin.

## References

[1] Sorokin I Mamoulakis C Miyazawa K Rodgers A Talati J Lotan Y. Epidemiology of stone disease across the world. World J Urol. (2017) 35:1301–20. 10.1007/s00345-017-2008-6 28213860

[2] Assimos D Krambeck A Miller NL Monga M Murad MH Nelson CP. Surgical management of stones: American urological association/endourological society guideline, PART I. J Urol. (2016) 196:1153–60. 10.1016/j.juro.2016.05.090 27238616

[3] Skolarikos A Gross AJ Krebs A Unal D Bercowsky E Eltahawy E. Outcomes of flexible ureterorenoscopy for solitary renal stones in the CROES URS global study. J Urol. (2015) 194:137–43. 10.1016/j.juro.2015.01.112 25676432

[4] Kramolowsky E McDowell Z Moore B Booth B Wood N. Cost analysis of flexible ureteroscope repairs: evaluation of 655 procedures in a community-based practice. J Endourol. (2016) 30:254–6. 10.1089/end.2015.0642 26542761

[5] Semins MJ George S Allaf ME Matlaga BR. Ureteroscope cleaning and sterilization by the urology operating room team: the effect on repair costs. J Endourol. (2009) 23:903–5. 10.1089/end.2008.0489 19445639

[6] Davis NF Quinlan MR Browne C Bhatt NR Manecksha RP D'Arcy FT. Single-use flexible ureteropyeloscopy: a systematic review. World J Urol. (2018) 36:529–36. 10.1007/s00345-017-2131-4 29177820

[7] Mager R Kurosch M Höfner T Frees S Haferkamp A Neisius A. Clinical outcomes and costs of reusable and single-use flexible ureterorenoscopes: a prospective cohort study. Urolithiasis. (2018) 46:587–93. 10.1007/s00240-018-1042-1 29356873

[8] Hennessey DB Fojecki GL Papa NP Lawrentschuk N Bolton D. Single-use disposable digital flexible ureteroscopes: an *ex vivo* assessment and cost analysis. BJU Int. (2018) 121(Suppl. 3):55–61. 10.1111/bju.14235 29656467

[9] Martin CJ McAdams SB Abdul-Muhsin H Lim VM Nunez-Nateras R Tyson MD. The economic implications of a reusable flexible digital ureteroscope: a cost-benefit analysis. J Urol. (2017) 197:730–5. 10.1016/j.juro.2016.09.085 27693449

[10] Ventimiglia E Somani BK Traxer O. Flexible ureteroscopy: reuse? or is single use the new direction? Curr Opin Urol. (2020) 30:113–9. 10.1097/MOU.0000000000000700 31815748

[11] Fernbach SK Maizels M Conway JJ. Ultrasound grading of hydronephrosis: introduction to the system used by the Society for Fetal Urology. Pediatr Radiol. (1993) 23:478–80. 10.1007/BF02012459 8255658

[12] Riccabona M Avni FE Blickman JG Dacher JN Darge K Lobo ML. Imaging recommendations in paediatric uroradiology: minutes of the ESPR workgroup session on urinary tract infection, fetal hydronephrosis, urinary tract ultrasonography and voiding cystourethrography, Barcelona, Spain, June 2007. Pediatr Radiol. (2008) 38:138–45. 10.1007/s00247-007-0695-7 18071685

[13] Dindo D Demartines N Clavien PA. Classification of surgical complications: a new proposal with evaluation in a cohort of 6336 patients and results of a survey. Ann Surg. (2004) 240:205–13. 10.1097/01.sla.0000133083.54934.ae 15273542 PMC1360123

[14] Singer M Deutschman CS Seymour CW Shankar-Hari M Annane D Bauer M. The third international consensus definitions for sepsis and septic shock (Sepsis-3). JAMA. (2016) 315:801–10. 10.1001/jama.2016.0287 26903338 PMC4968574

[15] Smith A Averch TD Shahrour K Opondo D Daels FP Labate G. A nephrolithometric nomogram to predict treatment success of percutaneous nephrolithotomy. J Urol. (2013) 190:149–56. 10.1016/j.juro.2013.01.047 23353048

[16] Doizi S Kamphuis G Giusti G Andreassen KH Knoll T Osther PJ. First clinical evaluation of a new single-use flexible ureteroscope (LithoVue™): a European prospective multicentric feasibility study. World J Urol. (2017) 35:809–18. 10.1007/s00345-016-1936-x 27671898

[17] Qi S Yang E Bao J Yang N Guo H Wang G. Single-use versus reusable digital flexible ureteroscopes for the treatment of renal calculi: a prospective multicenter randomized controlled trial. J Endourol. (2020) 34:18–24. 10.1089/end.2019.0473 31432716

[18] Kam J Yuminaga Y Beattie K Ling KY Arianayagam M Canagasingham B. Single use versus reusable digital flexible ureteroscopes: a prospective comparative study. Int J Urol. (2019) 26:999–1005. 10.1111/iju.14091 31448473

[19] Usawachintachit M Isaacson DS Taguchi K Tzou DT Hsi RS Sherer BA. A prospective case-control study comparing lithovue, a single-use, flexible disposable ureteroscope, with flexible, reusable fiber-optic ureteroscopes. J Endourol. (2017) 31:468–75. 10.1089/end.2017.0027 28287823 PMC5439446

[20] Mitchell S Havranek E Patel A. First digital flexible ureterorenoscope: initial experience. J Endourol. (2008) 22:47–50. 10.1089/end.2007.0046 18315473

[21] Somani BK Al-Qahtani SM de Medina SD Traxer O. Outcomes of flexible ureterorenoscopy and laser fragmentation for renal stones: comparison between digital and conventional ureteroscope. Urology. (2013) 82:1017–9. 10.1016/j.urology.2013.07.017 24001703

[22] Salvadó JA Cabello JM Moreno S Cabello R Olivares R Velasco A. Endoscopic treatment of lower pole stones: is a disposable ureteroscope preferable? results of a prospective case-control study. Central Eur J Urol. (2019) 72:280–4. 10.5173/ceju.2019.1962 31720031 PMC6830478

[23] Marchini GS Torricelli FC Batagello CA Monga M Vicentini FC Danilovic A. A comprehensive literature-based equation to compare cost-effectiveness of a flexible ureteroscopy program with single-use versus reusable devices. Int Braz J Urol. (2019) 45:658–70. 10.1590/s1677-5538.ibju.2018.0880 31397987 PMC6837614

[24] Li Y Chen J Zhu Z Zeng H Zeng F Chen Z. Comparison of single-use and reusable flexible ureteroscope for renal stone management: a pooled analysis of 772 patients. Transl Androl Urol. (2021) 10:483–93. 10.21037/tau-20-1009 33532336 PMC7844498

[25] Salvadó JA Olivares R Cabello JM Cabello R Moreno S Pfeifer J. Retrograde intrarenal surgery using the single–use flexible ureteroscope Uscope 3022 (Pusen™): evaluation of clinical results. Central Eur J Urol. (2018) 71:202–7. 10.5173/ceju.2018.1653 30038811 PMC6051368

[26] Dessyn JF Balssa L Chabannes E Jacquemet B Bernardini S Bittard H. Flexible ureterorenoscopy for renal and proximal ureteral stone in patients with previous ureteral stenting: impact on stone-free rate and morbidity. J Endourol. (2016) 30:1084–8. 10.1089/end.2016.0045 27527667

[27] Wang F Yang Y Chen H Huang H Huang W Weng Z. The application of a single-use fiberoptic flexible ureteroscope for the management of upper urinary calculi. Int Urol Nephrol. (2018) 50:1235–41. 10.1007/s11255-018-1895-9 29797215

[28] Legemate JD Kamphuis GM Freund JE Baard J Oussoren HW Spijkerman IJB. Pre-use ureteroscope contamination after high level disinfection: reprocessing effectiveness and the relation with cumulative ureteroscope use. J Urol. (2019) 201:1144–51. 10.1097/JU.0000000000000108 30707130

[29] Taguchi K Usawachintachit M Tzou DT Sherer BA Metzler I Isaacson D. Micro-costing analysis demonstrates comparable costs for lithovue compared to reusable flexible fiberoptic ureteroscopes. J Endourol. (2018) 32:267–73. 10.1089/end.2017.0523 29239227

[30] Molina W Warncke J da Silva RD Gustafson D Nogueira L Kim F. Cost analysis of utilization of disposable flexible ureteroscopes in high risk for breakage cases. J. Urol. (2018) 199:e1047. 10.1016/j.juro.2018.02.2499

